# Food Safety: Old Habits, New Perspectives

**DOI:** 10.3201/eid1306.070310

**Published:** 2007-06

**Authors:** Charles Higgins

**Affiliations:** * National Park Service Public Health Division, Washington, DC, USA

Anyone who works in food safety sooner or later discovers that one of the most valuable tools for prevention is simply reading about and understanding how past outbreaks have occurred. Using major and frequently famous or at least newsworthy outbreaks, Phyllis Entis in Food Safety: Old Habits, New Perspectives illustrates how critical factors come together to produce tragic and largely preventable results. This nicely written reference book reads more like an engaging novel in some ways, complete with bad guys (pathogens and sometimes careless corporations) and good guys (intrepid and resourceful outbreak investigators). The author’s unique style, usually avoided in science writing but appropriately used here, tells the tale of modern food safety issues so well that the book, literally, is difficult to put down.

Each of the 17 chapters covers a different food safety principle, illuminating how modern microbes often team up with old practices, short-sighted decisions, or current consumer trends to produce an outbreak. Chapters conclude with a concise “lessons learned” summary, such as this conclusion from Chapter 3: “Whether it’s serotype Enteritidis in eggs or *C. botulinum* in eggplant, the challenge is the same. Recipes that do not include an adequate final cooking step have become increasingly popular with consumers and can be a significant source of food-borne illness.”

One of the few downsides of this book is that it does leave the reader with the somewhat sensational impression that most food businesses are out to get the consumer. While the examples of greed and negligence are true, positive examples of good corporate behavior could have illustrated prevention and better balanced the portrayal of the food industry. Despite this small drawback, the tables are “one-stop shopping” for anyone looking for lists of outbreaks, and the fact boxes inserted here and there provide marvelous tidbits of information. A “who’s who” of microbes at the end of the book is an added bonus.

Whether someone is preparing to teach a food safety course, looking for information about how and why outbreaks occur, or trying to get the facts on a critical food safety event, this author has already done all of the homework. For any food safety professional who has ever dreamed of the ultimate literature search, the references at the end of each chapter are breathtaking. This book is a must-have for any serious food safety professional.

